# Restricted Kinematic Alignment, the Fundamentals, and Clinical Applications

**DOI:** 10.3389/fsurg.2021.697020

**Published:** 2021-07-20

**Authors:** Pascal-André Vendittoli, Sagi Martinov, William G. Blakeney

**Affiliations:** ^1^Department of Surgery, CIUSSS-de-L'Est-de-L'Ile-de-Montréal, Hôpital Maisonneuve Rosemont, Montréal, QC, Canada; ^2^Department of Surgery, Université de Montréal, Montréal, QC, Canada; ^3^Clinique Orthopédique Duval, Laval, QC, Canada; ^4^Personalized Arthroplasty Society, Montréal, QC, Canada; ^5^Department of Orthopedic Surgery, Royal Perth Hospital, Perth, WA, Australia

**Keywords:** knee-surgery, alignment, kinematic, personalized medicine, anatomical, arthroplasty (replacement), mechanical, restricted

## Abstract

**Introduction:** After a better understanding of normal knee anatomy and physiology, the Kinematic Alignment (KA) technique was introduced to improve clinical outcomes of total knee arthroplasty (TKA). The goal of the KA technique is to restore the pre-arthritic constitutional lower limb alignment of the patient. There is, however, a large range of normal knee anatomy. Unusual anatomies may be biomechanically inferior and affect TKA biomechanics and wear patterns. In 2011, the leading author proposed the restricted kinematic alignment (rKA) protocol, setting boundaries to KA for patients with an outlier or atypical knee anatomy.

**Material and Equipment:** rKA aims to reproduce the constitutional knee anatomy of the patient within a safe range. Its fundamentals are based on sound comprehension of lower limb anatomy variation. There are five principles describing rKA: (1) Combined lower limb coronal orientation should be ± 3° of neutral; (2) Joint line orientation coronal alignment should be within ± 5° of neutral; (3) Natural knee's soft tissues tension/ laxities should be preserved/restored; (4) Femoral anatomy preservation is prioritized; (5) The unloaded/most intact knee compartment should be resurfaced and used as the pivot point when anatomical adjustment is required. An algorithm was developed to facilitate the decision-making.

**Methods:** Since ~50% of patients will require anatomic modification to fit within rKA boundaries, rKA is ideally performed with patient-specific instrumentation (PSI), intra-operative computer navigation or robotic assistance. rKA surgical technique is presented in a stepwise manner, following the five principles in the algorithm.

**Results:** rKA produced excellent mid-term clinical results in cemented or cementless TKA. Gait analysis showed that rKA TKA patients had gait patterns that were very close to a non-operated control group, and these kinematics differences translated into significantly better postoperative patient-reported scores than mechanical alignment (MA) TKA cases.

**Discussion:** Aiming to improve the results of MA TKA, rKA protocol offers a satisfactory compromise that recreates patients' anatomy in most cases, omitting the need for extensive corrections and soft tissue releases that are often required with MA. Moreover, it precludes the reproduction of extreme anatomies seen with KA.

## Introduction

### Mechanical Alignment Limitations

Most patients following mechanically aligned (MA) total knee arthroplasty (TKA) do not report a natural joint (1). Every fifth patient is dissatisfied (2), every second patient presents residual symptoms (3), and every fourth one would not undergo the same surgery again (4). Following TKA, patients walk with a diminished total range of knee motion and significant kinematic discrepancies, as demonstrated by gait analysis studies (5). Historically, TKA implantation lacked instrument precision, and technical errors were frequent. Surgeons focused mainly on implant survivorship rather than on recreating a normal knee function (6). To ensure satisfactory prosthetic survivorship, the mechanical alignment (MA) technique was introduced. Neutral femoral and tibial cuts with adjusted femoral rotation and ligamentous releases to create equal flexion and extension gaps were the foundation of this simple method of knee alignment. This “one size fits all” philosophy, albeit reproducible, does not incorporate the full range of normal knee anatomy (7).

**Figure 1:**
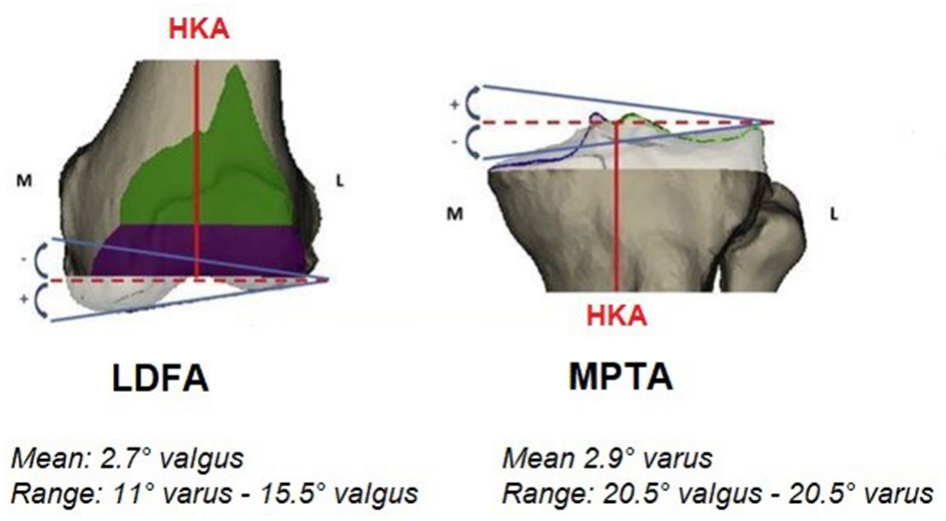
Anatomic modification linked to mechanical alignment technique on the distal femur and proximal tibia.

Even though the mean hip-knee-ankle angle (HKA) is close to neutral, in a study of 4,884 lower limb CT-scans of patients scheduled for TKA, we found that only 0.1% of patients had both a mechanical proximal tibial angle (mPTA) and mechanical distal femoral angle (mDFA) at neutral, which is MA goal. Such modifications of the bony anatomy will affect the soft tissue laxities and knee balance (Figure 1).

Simulating MA bone cuts on 1,000 knee CT scans, we found that MA results in many cases of gap asymmetries (8, 9). Mediolateral imbalances of more than 3 mm were observed in 25 and 54% of varus and valgus knees, respectively. Only 49% of varus and 18% of valgus knees had <3 mm of imbalance in mediolateral and flexion/extension gaps when employing trans-epicondylar axis for femoral rotation. Some imbalances might not be surgically correctable and may result in residual instability and poor results in knee replacement patients. A better understanding of normal knee joint functional anatomy led to the introduction of Kinematic Alignment (KA) technique to improve clinical outcomes after knee arthroplasty. It intends to restore the patient's pre-arthritic constitutional lower limb alignment and the orientation of its joint surfaces. The KA TKA technique is a joint resurfacing procedure rarely requiring soft tissue release (10–12). On the other hand, concerns remain about restoring “outlier” or “pathological” anatomies, which may be incompatible with current TKA prostheses and fixation methods.

### Are All Observed Anatomies Physiologic?

The human knee anatomy is highly variable, and pathological changes increase this fluctuation even further (7, 13, 14). In 4,884 lower limb CT-scans of patients scheduled for TKA: arithmetic HKA (aHKA) was >3° in 40%, >5° in 19%, and >10° in 3% (7). The mDFA mean was 2.7° valgus, ranging from 11° varus to 15.5° valgus. The mPTA mean was 2.9° varus within 20.5° varus and 20.5° valgus range. This large spectrum of mPTA and mDFA values exhibits the vast variability in patient anatomy. The more outlying alignment may be inherently biomechanically inferior. It may have been altered by different conditions that might expedite the degenerative changes, such as trauma, developmental deformity, tumors or previous surgery.

**Figure 2:**
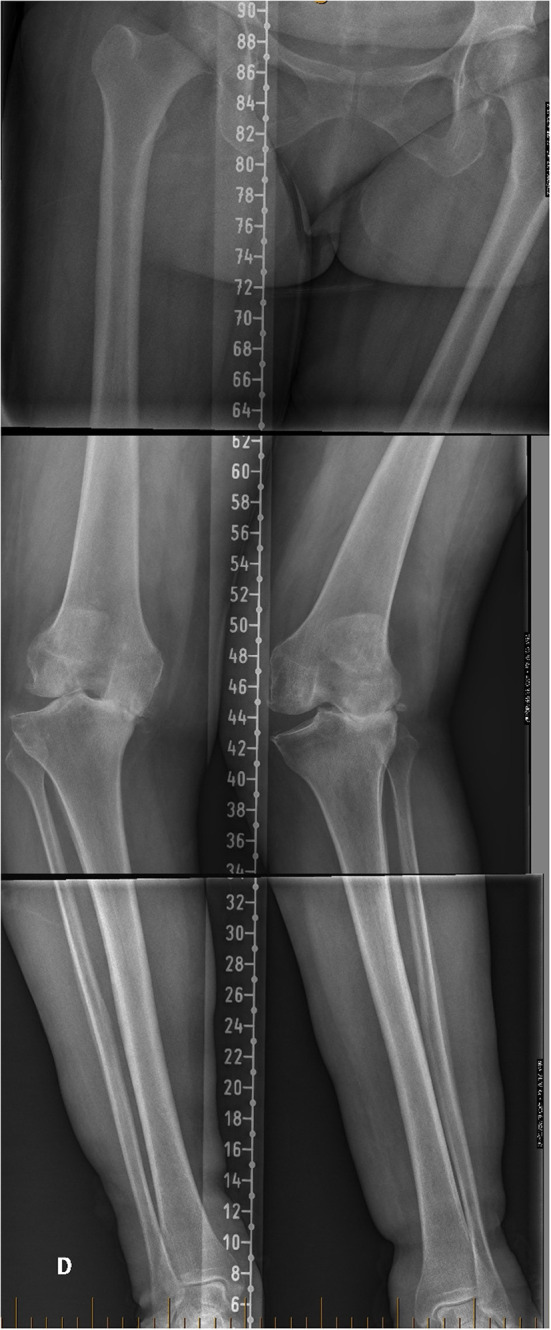
Lower limb full-length radiographs showing lower limbs with windswept deformity. Her mechanical distal femoral angle (mDFA) is 10° valgus on the right femur vs. 1° on the left side. Regarding tibial anatomy, her right mechanical proximal tibial angle (mPTA) is at 0° vs. 5° varus on the left. Because of her important lower limb asymmetry, we consider her lower limb anatomy to be pathologic. Applying unrestricted KA technique would reproduce her lower limbs malalignment.

**Figure 3:**
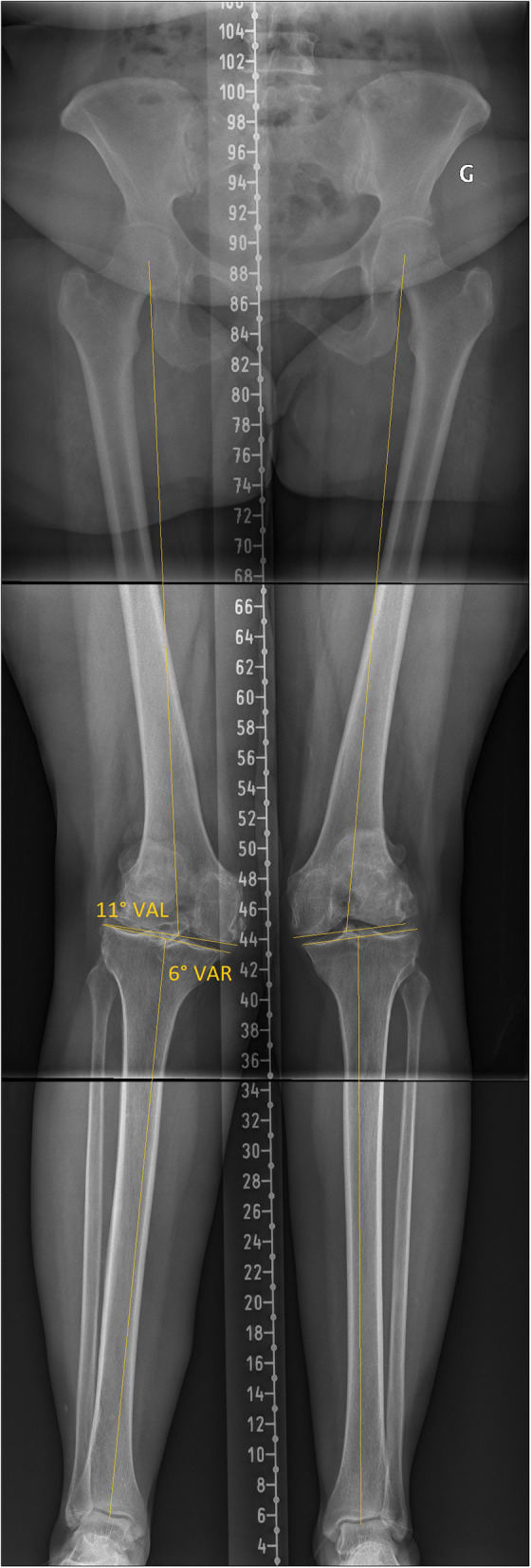
Lower limb full-length radiographs show bilateral valgus lower limbs with severe right knee OA. Her mDFA is 11° valgus and her mPTA is 6° varus. Applying unrestricted KA technique would reproduce her right lower limb alignment, reproducing extreme joint orientation (obliquity) and a lower limb arithmetic HKA (aHKA) in 5° of valgus. Such outlier anatomy might not be compatible with current TKA implant bearing and fixation methods.

The existence of patho-anatomies can be demonstrated by their unilateral occurrence or bilateral asymmetry in some patients (Figures 2, 3). The surgeon should not blindly reproduce the identical anatomy of outlier patients as it might negatively affect the TKA biomechanics and increase wear. On the other hand, these extreme cases are the most impacted by a MA technique as it significantly modifies their anatomy and likely causes soft tissue imbalances, variation of the femoral flexion axis, changes in joint line orientation, and alteration of knee kinematics.

A computer simulation study comparing the effects of MA or KA on a single knee joint replacement model showed that KA TKA produced near-normal knee kinematics, including higher femoral rollback and external rotation of the femoral component (15). However, it demonstrated increased contact stresses, questioning long-term results. A study of 178 MA knee arthroplasty revisions found that knees with higher varus alignment had greater total damage of the retrieved polyethylene components (16). They also demonstrated that MA-TKAs tended to drift away from a neutral mechanical alignment toward the preoperative varus deformity. Other clinical and simulator studies have found an association between polyethylene wear and varus alignment (17–19). Tibial baseplate migration and greater tibial varus were weakly correlated (*r*^2^ = 0.45) in a study with a 10-year follow-up (20). Interestingly, baseplate migration was not affected by overall limb alignment (HKA of 1.3° valgus to over 10° varus). No difference was shown between those within ± 3° of neutral and those higher than 3°. These studies suggest that systematic replicating the patho-anatomy of all patients might not be suitable for survivorship of the TKA using current materials and fixation methods.

## Fundamentals of Restricted Kinematic Alignment

### Five rKA Principles

The rKA protocol has been developed as an alternative to the unrestricted KA proposed by Howell (11, 21) for patients with an outlier or atypical knee anatomy. The concept of rKA aims to reproduce patient's constitutional knee anatomy within a safe range while avoiding extreme or pathological anatomies that have been demonstrated to exist (7). The five principles to perform rKA TKA are explained by its designer (PAV) in Video 1. https://youtu.be/k6qdpyh80Tc

### rKA Principle 1: HKA Boundaries

**Figure 4:**
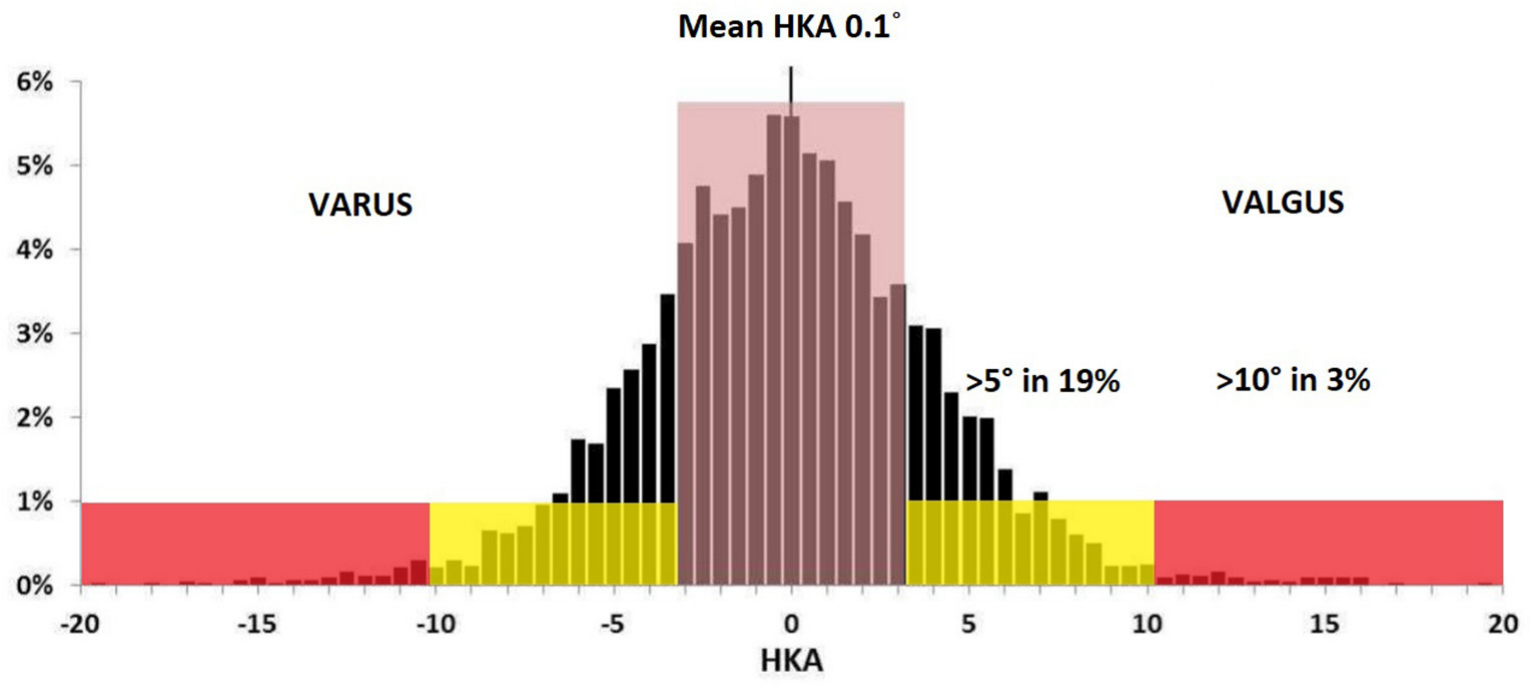
Lower limb Ct-Scan, preoperative aHKA distribution from a population of 4,884 patients scheduled for a TKA (7).

Historically, the hip-knee-ankle (HKA) angle serves as a reference for knee alignment. MA TKA data has demonstrated that the survivorship of the implant is not affected if the values of HKA are kept within 3° (22). In a population of 4,884 patients awaiting a TKA, a total of 40% of patients had an aHKA >3°, and 3% had extreme anatomy with an HKA of >10° in varus or valgus (7) (Figure 4). Aiming at reproducing individual lower limb anatomy while keeping aHKA within ± 3° range establishes the first rKA principle.

### rKA Principle 2: Joint Line Orientation

**Figure 5:**
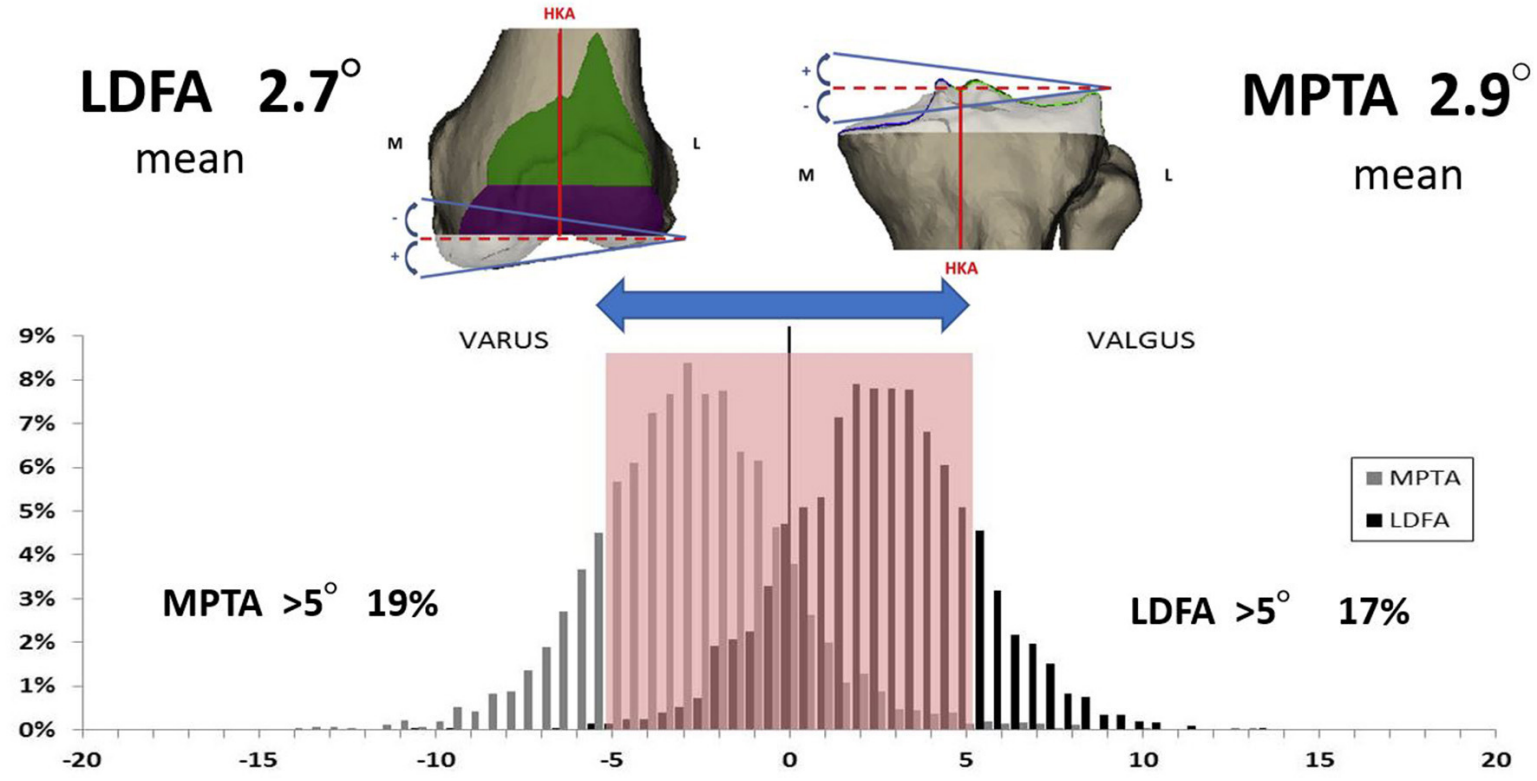
Lower limb Ct-Scan preoperative mDFA and mPTA distribution from a population of 4,884 patients scheduled for a TKA (7).

It is very rare (0.1%) for the human knee to have a neutral joint line (neutral mDFA + neutral mPTA) (7). In fact, the mean mPTA is in 2.9° varus, and the mean mLDFA is in 2.7° valgus. Furthermore, we found that 80% had an mDFA and mPTA below 5° (Figure 5). Keeping in mind the mean values and aiming to include the vast majority of patients, the second rKA principle strives to reproduce individual anatomy while keeping the LDFA and the mPTA within ± 5°. By this second principle, rKA limits the joint obliquity to 5°. Selecting 5° also includes all patients with ± 2° from the mean values (80% of the population).

Applying the first two rKA principles, 51% of the population would undergo a classic KA without any modification. Another 30% would have a correction of <1° (mean tibia 0.5° and mean femur 0.3°). The remaining 20% of patients would require more substantial adjustments (7). Awaiting further evidence regarding the acceptable limb alignment boundaries, the proposed rKA boundaries are sound and reasonable but may evolve with time.

### rKA Principle 3: Preservation of Soft Tissue Laxities

The third rKA principle is to preserve/restore the natural knee's soft tissue tension. Physiologic soft tissue laxities, including ligaments, tendon and capsular structures, play a key role in knee kinematics. It has been shown that collateral ligaments are not isometric, and their laxities change over the arc of motion. The medial collateral ligament (MCL) is tighter than the lateral collateral ligament (LCL), and both ligaments are tighter in extension than in flexion (23). Furthermore, ligamentous laxity is higher in females than in the male population, and the inter-individual variation is wide. Following this notion, MA or any other technique that includes ligaments tensioning or gap balancing aiming to create an equal tension of medial and lateral collateral structures does not restore the correct kinematics of the knee (24, 25). With rKA, soft tissue release should only be performed in cases outside the boundaries of principles 1 and 2; i.e., a systematic deep MCL release at exposure should be avoided. In the senior author's experience, soft tissue releases are required when rKA boundaries necessitate an anatomy correction of more than 2-3°. For example, in a varus knee where the mPTA is modified from 8° to 5°, a deep MCL release should be enough to balance the gap modification in most cases.

### rKA Principle 4: Femoral Anatomy Preservation

**Figure 6:**
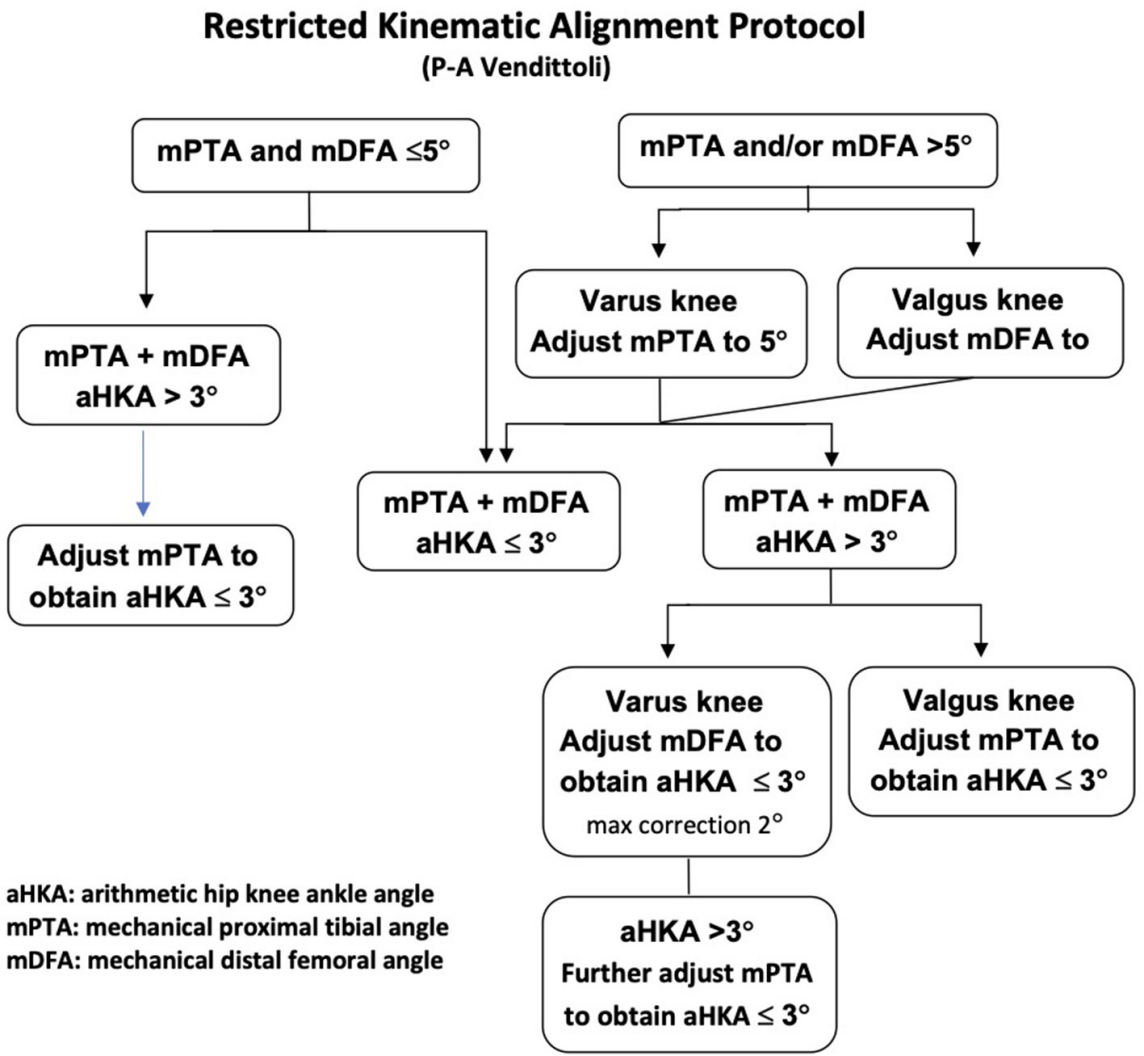
rKA algorithm by P-A Vendittoli.

In consensus with numerous scientists, we consider the femoral anatomy to be fundamental to knee kinematics (26–28). The fourth rKA principle states that in cases where the patient's anatomy is outside the rKA boundaries described in principles 1 and 2, femoral anatomy preservation is prioritized over the tibia. The rKA algorithm (Figure 6) advocates correcting the most contributing bone to the alignment's deviation. In most mild varus knees, the tibia is the main contributor, whereas it is the femur in valgus cases. In more extreme cases (e.g., aHKA>10°), both the femur and the tibia contribute to the outlying anatomy (i.e., severe varus with the femur and tibia in varus). In such varus cases, we limit the femoral anatomy modification to 2°, and in severe valgus cases, after reducing the mDFA to 5°, no further modification to the femur is added. The tibia will have to be in 2° of varus to keep the overall aHKA within ± 3° (Principle 1).

**Figure 7:**
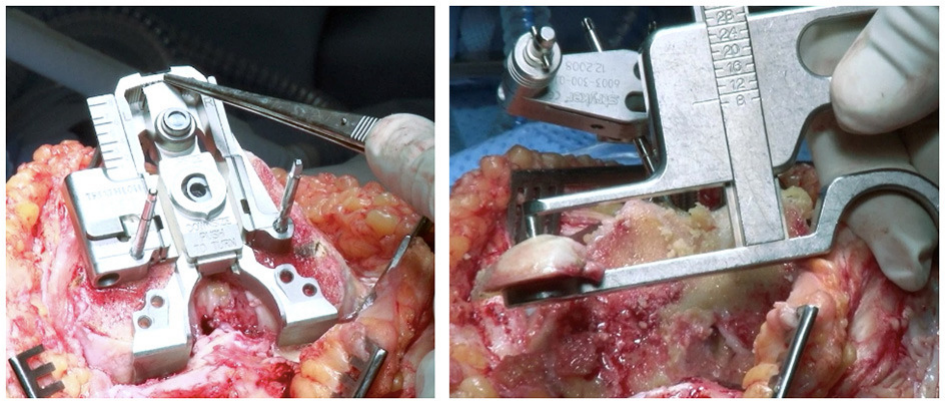
Intraoperative photograph of posterior referencing guide placed in neutral femoral rotation (left). The resected posterior femoral condyle measurement shows 9 mm corresponding to femoral component thickness (right).

Furthermore, no external femoral rotation is set when using the rKA protocol. For posterior condyles resurfacing, a posterior referencing guide is set to neutral rotation, thus resecting only the implant thickness of the posterior condyles matching each patient's native femoral orientation (Figure 7).

In cases where tibial anatomy is modified, e.g., mPTA of 8° is reduced to 5°, both the extension and flexion gaps will be affected (tightening), MCL release should be performed to restore the mediolateral gap balance in both extension and flexion. As described above, rKA does not aim toward MCL/LCL isometry since it would compromise natural knee kinematics, neither does it modify femoral bone cuts to create balanced gaps (as in gap balancing or functional alignment techniques).

### rKA Principle 5: Pivot Point

**Figure 8:**
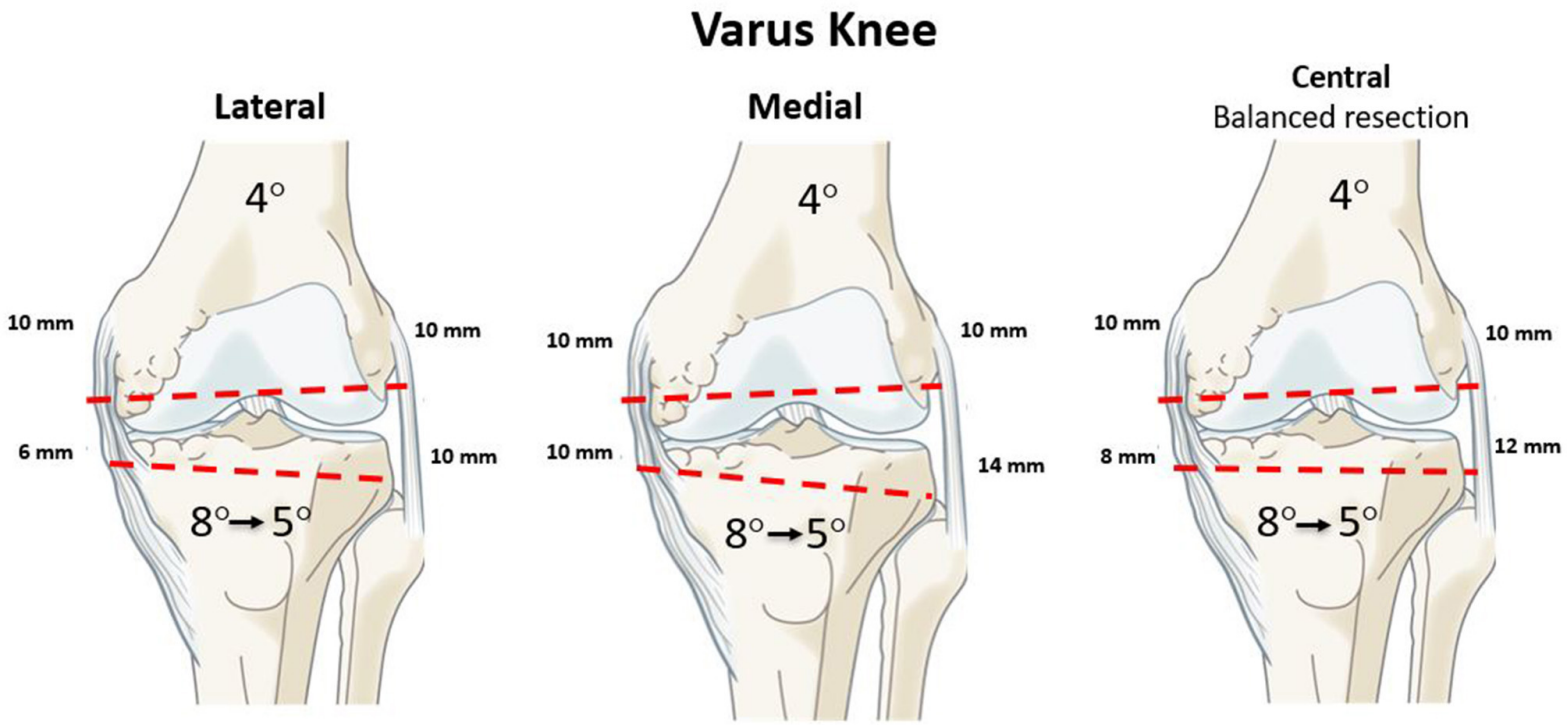
An example of varus knee with mDFA 4° valgus, mPTA 8° varus and aHKA 4° varus. Following rKA principles, the mPTA is adjusted to 5°, consequently changing aHKA to 1° varus. Using a lateral pivot point (shown on the left), the intact (lateral) compartment is resurfaced to accommodate an implant thickness (10 mm), creating a tighter medial gap which might require a deep MCL release. The medial pivot (central image) would result in thicker bone resection of the intact compartment and enlarging the lateral gap. The right figure shows a balanced bone resection with a central pivot. It complexifies the decision-making and requires PSI or robotics.

**Figure 9:**
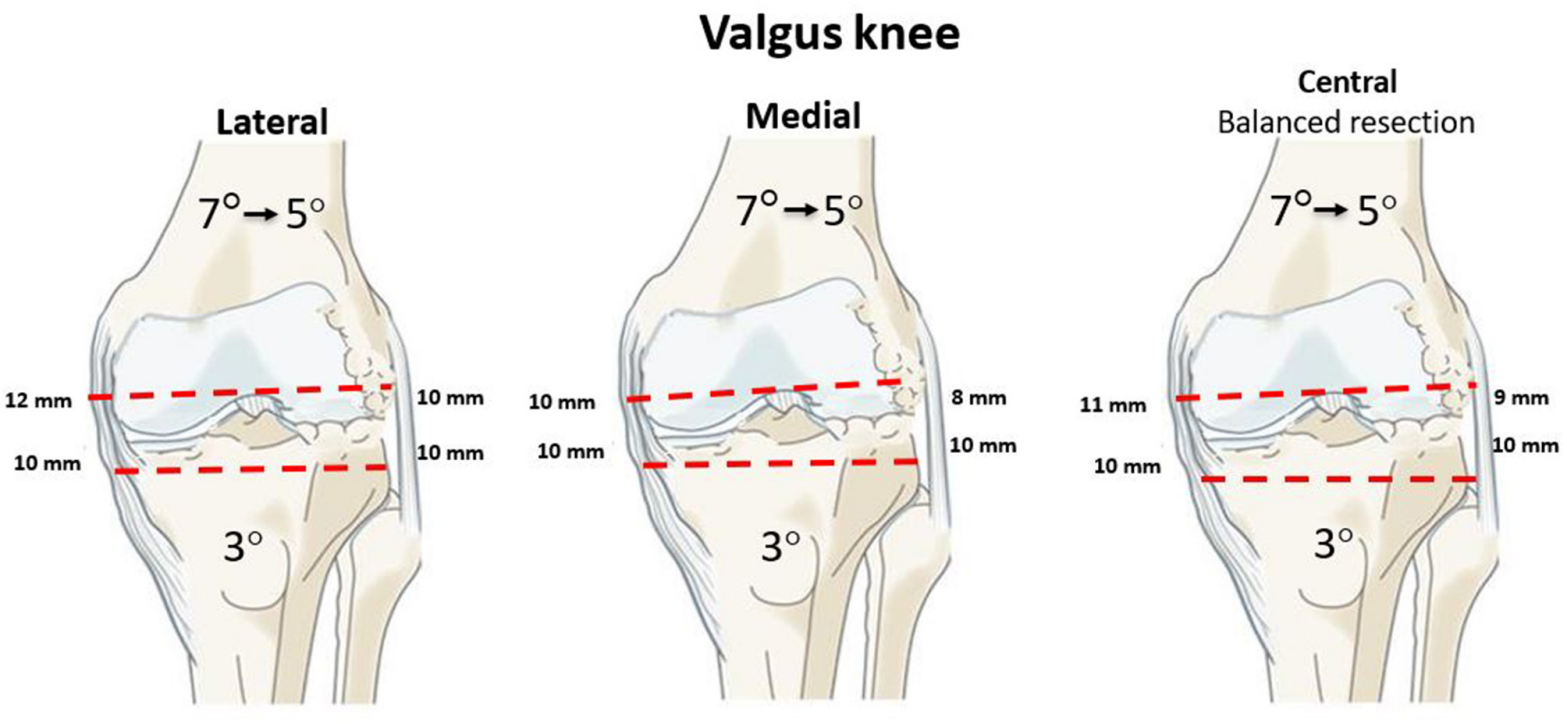
A valgus knee example with mDFA 7° valgus, mPTA 3° varus and aHKA 4° valgus. According to rKA protocol, aHKA should be ± 3°; thus mDFA is modified to 5° valgus. The left image presents a lateral pivot resulting in a 2 mm medial compartment imbalance, which may be detrimental for a valgus knee. The central image demonstrates the medial pivot creating tightness in the damaged lateral compartment while maintaining medial stability. The right figure shows a balanced bone resection with a central pivot. It complexifies the decision-making and requires PSI or robotics.

When outside the boundaries set in principles 1 and 2, the surgeon needs to decide where the anatomical modification (bone resection changing patient's anatomy) should be; medial, lateral or balanced on both sides. Cut orientation can be adjusted using 3 different pivot points: medial, central or lateral. A medial pivot point would resurface the medial compartment and modify the resection thickness on the lateral side (vice versa for a lateral pivot). A central pivot would change cut thicknesses on both compartments. The fifth rKA principle proposes to resurface (resection thicknesses equal to those of the implant) the unloaded knee compartment, and cut adjustment is performed on the worn side. This will modify the resection thickness on the worn side: medial in varus (Figure 8), lateral in valgus (Figure 9).

By following this principle, the intact compartment is resurfaced by cutting the exact thickness of bone matching the implant thickness, thus preserving the joint line level and preserving bone. As in MA, this leaves a tighter damaged compartment. Most surgeons will then feel comfortable performing the required ligamentous release as they would do with MA. A balanced resection with a central pivot is appealing but hard to manage without sophisticated preoperative planning or intra-operative automated decision tool.

### rKA Algorithm

To facilitate decision-making, an rKA algorithm following the five rKA principles is presented in Figure 6. As stated above, 51% of the patients present aHKA 3° and with both mPTA and mDFA ≤5°, implying that no adjustment is needed for those knee arthroplasties.

If the mPTA and mDFA are <5° but the aHKA is >3° varus (in 8% of the cases) or >3° valgus (in 7% of cases), then, following the fourth rKA principle of femoral anatomy preservation, the mPTA should be corrected to fall within 3° of aHKA. In cases with mPTA and/or mDFA >5° (right side of the algorithm), in varus knees, the mPTA needs to be adjusted to 5°; whilst in valgus knees, the mDFA should be brought to 5°. If those resections bring the aHKA ≤ 3°, the rKA objective is achieved. If the resultant aHKA is >3°, the previously unchanged parameter should be corrected, namely mDFA in varus knees and mPTA in valgus until the aHKA is ≤ 3°. In rare cases, where the above-mentioned steps do not lead to the desired aHKA ≤ 3°, MPTA should be further corrected. Ligamentous releases are rarely needed in cases with anatomic modifications of <3°. In more significant corrections, minimal releases can be done (usually, to a much lesser extent than in MA).

In our simulation study, unusual anatomy was observed in 17% of knees, with both the femur and tibia articular surfaces orientation in varus or valgus (7). As both bones contribute to the same overall HKA deviation, the surgeon has to decide which one to correct to reach the safe range. In our opinion, the femoral flexion axis plays a more significant role in knee kinematics; hence our practice maximally preserves femoral anatomy and performs most modifications of the tibia. For example, a valgus knee (aHKA 10° valgus) with a femur in 9° valgus and a tibia in 1° valgus, the femoral cut is modified to a maximum of 5° valgus and the tibial cut corrected to 2° varus, creating an aHKA of 3° valgus. Similarly, in severe varus knee (aHKA of 8° varus), with a femur 2° varus and tibia 6° varus, the femoral orientation is maintained (2° varus), and the tibial varus is reduced to from 6° to 1°, resulting in overall aHKA of 3° varus. It must be kept in mind that most of these cases have associated extra-articular deformities explaining these extreme alignments. The severe valgus is often due to diaphyseal tibia valga deformity, and the severe varus may be due to a femoral bowing creating this lower limb alignment (29). In these cases, KA (resurfacing the knee joint) will facilitate the preservation of ligament laxities but will not address the lower limb deviation caused by the extra-articular deformity. Performing the restricted KA protocol will correct the extra-articular deformity with intra-articular cuts and may require ligament adjustment to avoid secondary instability.

## Method: rKA Surgical Technique

In cases where the patient's anatomy fits into the rKA boundaries (51% of the cases), surgery could be performed using measured resection techniques with a caliper, as described by Howell (30). Doing so would require very meticulous preoperative radiographic planning to preserve the patient's anatomy. Since many patients will require anatomic modification to fit within rKA boundaries, rKA is ideally performed with patient-specific instrumentation (PSI), intra-operative computer navigation or robotic assistance (31). The following surgical technique applies to surgery with computer navigation or robotic assistance. A video is available for the PSI technique (Video 2) https://youtu.be/wKoSkbHmikI and computer navigation technique (Video 3) https://youtu.be/M8n-5l3Hzvo

After joint exposure, cartilage and bone loss thicknesses are estimated by comparing them to the intact areas. The intention is to restore the patient's pre-arthritic joint surfaces and lower limb alignment. For example, in a varus knee, for the unworn compartment (lateral), the distal femoral and proximal tibial cut resections are set at each implant's thickness (10 mm) (lateral pivot, rKA principle 5). Then, the cartilage wear thickness on the medial side of bone surfaces is assessed (intact cartilage = 0 mm, partial cartilage thickness wear = 1 mm, and exposed subchondral bone = 2 mm) (31). The cut angle is then adjusted to reach the desired medial resection thickness (e.g., a case with 2 mm of medial tibial wear (subchondral bone exposed), an 8-mm medial resection, and a 10-mm lateral resection should be performed.

Resections only differ from patient anatomy when the measured angles fall outside the pre-defined “safe range” as described in the rKA algorithm (rKA principles #1 and 2, Figure 6).

To preserve femoral anatomy (posterior and anterior joint surface orientations), the surgeon should aim to resurface the posterior condyles. Using a posterior referencing guide set to neutral rotation, the implant thickness on both posterior condyles will be ressected without modifying femoral rotation (Figure 7). Tibial component's rotation is set by its alignment with the femoral trial component, keeping the knee in 10 degrees of flexion. If the resected pieces do not match the computer plan or ligament laxities assessed with trial implants fall outside the expected native ligament laxity range (24), the resection accuracy can be confirmed by caliper measurement and cut adjustment is performed when needed (Figure 7).

## Clinical Results Using rKA

The rKA protocol aims to bring the extreme anatomies toward acceptable values by correcting the deformities and providing an implant orientation that is compatible with current materials and fixation methods. Comparing the required anatomy correction between MA and rKA in a cohort of 4,884 arthritic patients, significantly larger corrections were necessary with MA (7). The mean mPTA correction was 0.5° for rKA vs. 3.3° for MA (*p* < 0.001). Similarly, the mean mDFA correction was 0.3° for rKA vs. 3.2° for MA (*p* < 0.001). This highlights that MA introduces significant changes to normal anatomy. Consequently, these modifications of anatomy require larger soft tissue releases to balance the knee, which may have an unfavorable effect on normal biomechanics. The mediolateral and flexion-extension gap asymmetries were compared between measured resection MA and rKA protocol bone cut simulations in another study of 1,000 preoperative CT scans of patients awaiting knee arthroplasty. Greater than 2 mm extension space mediolateral (ML) imbalances occurred in 33% of TKA with MA technique vs. 8% of the knees with rKA. Imbalances of more than 4 mm were present in 11% of MA knees vs. 1% in rKA (*p* < 0.001). With MA, a higher rate of flexion space imbalance was created by the transepicondyar axis (TEA) technique (*p* < 0.001), compared to external rotation of 3° to posterior condyles (PC). rKA again performed better than both techniques (*p* < 0.001). MA with either TEA or PC, only 49 and 63% of the knees, respectively, had < 3 mm of imbalance throughout the extension and flexion spaces and medial and lateral compartments, vs. 92% with rKA (*p* < 0.001). A wide spectrum of complex collateral ligament imbalances, incorrigible by collateral ligament releases, caused by the significant anatomical modifications inherent in the MA technique, has been reported in the literature (7, 32).

A clinical series of the first 100 cemented TKA patients operated on using the rKA protocol demonstrated satisfactory functional outcomes at 2.4 years follow-up (33). Minor ligamentous releases were required in only 5% of the knees. Another study presented 100 cementless TKAs operated on using rKA protocol without any revision for aseptic loosening at 49 (32–60) months of follow-up (34). It also demonstrated excellent osseointegration of the implants, both on radiographic evaluation and on direct examination of implants in three revised patients- one following trauma that caused a tibial implant shift, one due to deep infection at 21 months after the index TKA, and one for instability due to implant under-sizing. The WOMAC, KOOS, and Forgotten Joint scores reported in this study were similar to those reported for cemented KA TKAs.

A study comparing the gait patterns of patients operated on with rKA vs. MA techniques found that the rKA TKA kinematics were significantly closer to healthy controls than in MA TKAs (33). When comparing the MA and control (healthy) groups, the former displayed a decreased maximum flexion (52° vs. 58°, *p* = 0.002), less sagittal plane range of motion (49° vs. 54°, *p* = 0.020) and increased adduction angle (2.0–7.5° vs. −2.8–3.0°, *p* < 0.05). KA group presented a significantly higher postoperative KOOS score when compared to the MA group (74 vs. 61, *p* = 0.034).

## Discussion: rKA vs. True KA, A Compromise?

Many surgeons are concerned about allowing too much varus or valgus with the KA technique. Howell et al. (35) reported 97.5% implant survivorship in a cohort of 222 KA TKAs at 10-year follow-up. There was no increased failure rate in patients with greater varus. The radio-stereometric analysis of TKAs randomized to MA or KA did not detect differences in implant migration between groups (31). There are few long-term follow-up studies on KA knee replacement, whereas MA TKAs have a long history of good survivorship (36–38). A mechanical axis ±3° of neutral has demonstrated better functional outcomes than TKAs with more extreme values (39–41). Increased rates of aseptic loosening related to malaligned components have been published (22, 42, 43). However, more recent studies have failed to demonstrate better survivorship or functional outcomes in prostheses aligned within ± 3° of neutral compared to malaligned ones (44–47). Caution should be taken, not transposing the results of these studies to KA. It is important to understand that an accurate KA, aiming for an HKA other than neutral, is inherently different from a failed MA TKA, targeting a neutral alignment. There are other factors than coronal alignment that affect the dynamic loading of the prosthetic knee. The joint line tends to remain parallel to the ground when standing, despite a range in alignment, in studies of both healthy, asymptomatic knees and in kinematic TKA patients (48, 49). The resultant functional joint line orientation may be favorable for the overall load profile of the prosthetic joint.

Some authors advise against widespread adoption of the KA technique due to the lack of long-term studies of KA TKAs (50). We believe the rKA protocol is an appealing compromise that allows the restoration of normal patient anatomy in the majority of cases. It averts the excessive corrections and ligamentous releases often required with MA but precludes the extremes of implant positioning that can be seen in unrestricted KA technique.

## Case Illustrations

### Case Example 1

**Figure 10:**
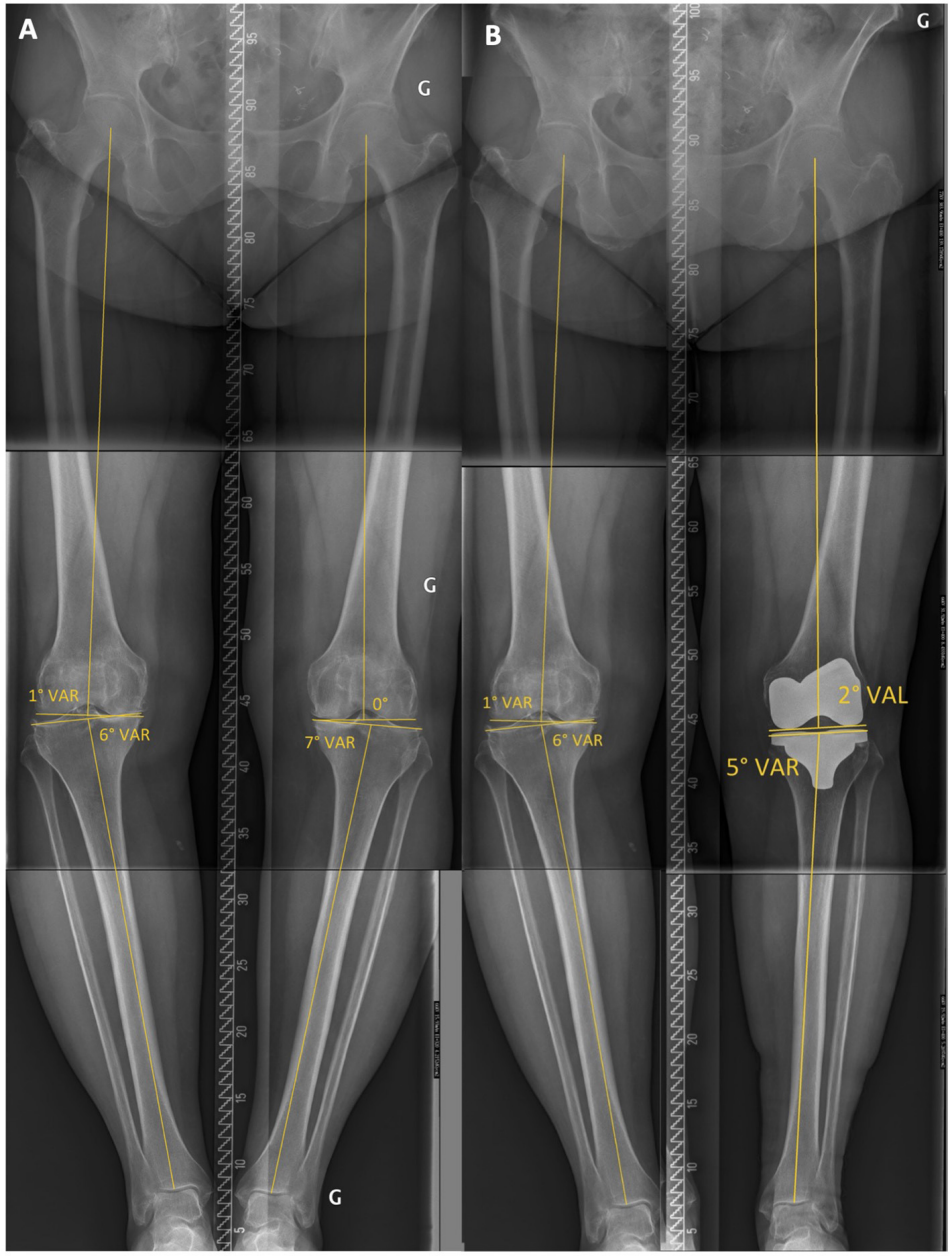
Case example 1, **(A)** preoperative standing long radiograph of a patient with a left lower limb mDFA of 0° and a mPTA of 7° varus, resulting in an aHKA of 7° varus. **(B)** on the postoperative radiograph, following the rKA algorithm, the implants mDFA is 2° valgus and mPTA is 5° varus.

A 62-year-old female with bilateral symptomatic varus knee osteoarthritis (Figure 10A). A preoperative radiograph shows a left lower limb mDFA of 0° and mPTA of 7° varus, resulting in an aHKA of 7° varus. Following the algorithm (Figure 6), we should correct the mPTA from 7° to 5° (Principle 2). Her neutral mDFA, would lead to an aHKA of 5°, which is >3° (Principle 1). Further tibial varus reduction (down to 3°) would impact the knee's flexion gaps balance. Instead, following the algorithm, we suggest adding 2° of valgus to the distal femoral cut. No femoral rotation modification is needed, and femoral anatomy modifications are minimized (Principle 4). The overall angle correction was 4° (2 on de femoral side and 2 on the tibial side). We resurfaced the lateral compartment (lateral pivot point, Principle #5) and reduced medial compartment resection thicknesses (~2 mm thinner cuts on both the femur and tibia). To achieve medio-lateral ligament balance, we had to perform a deep MCL release alone. The postoperative radiograph confirms the achievement of our goals with a mDFA of 2° valgus and a mPTA of 5° varus (Figure 10B).

### Case Example 2

**Figure 11:**
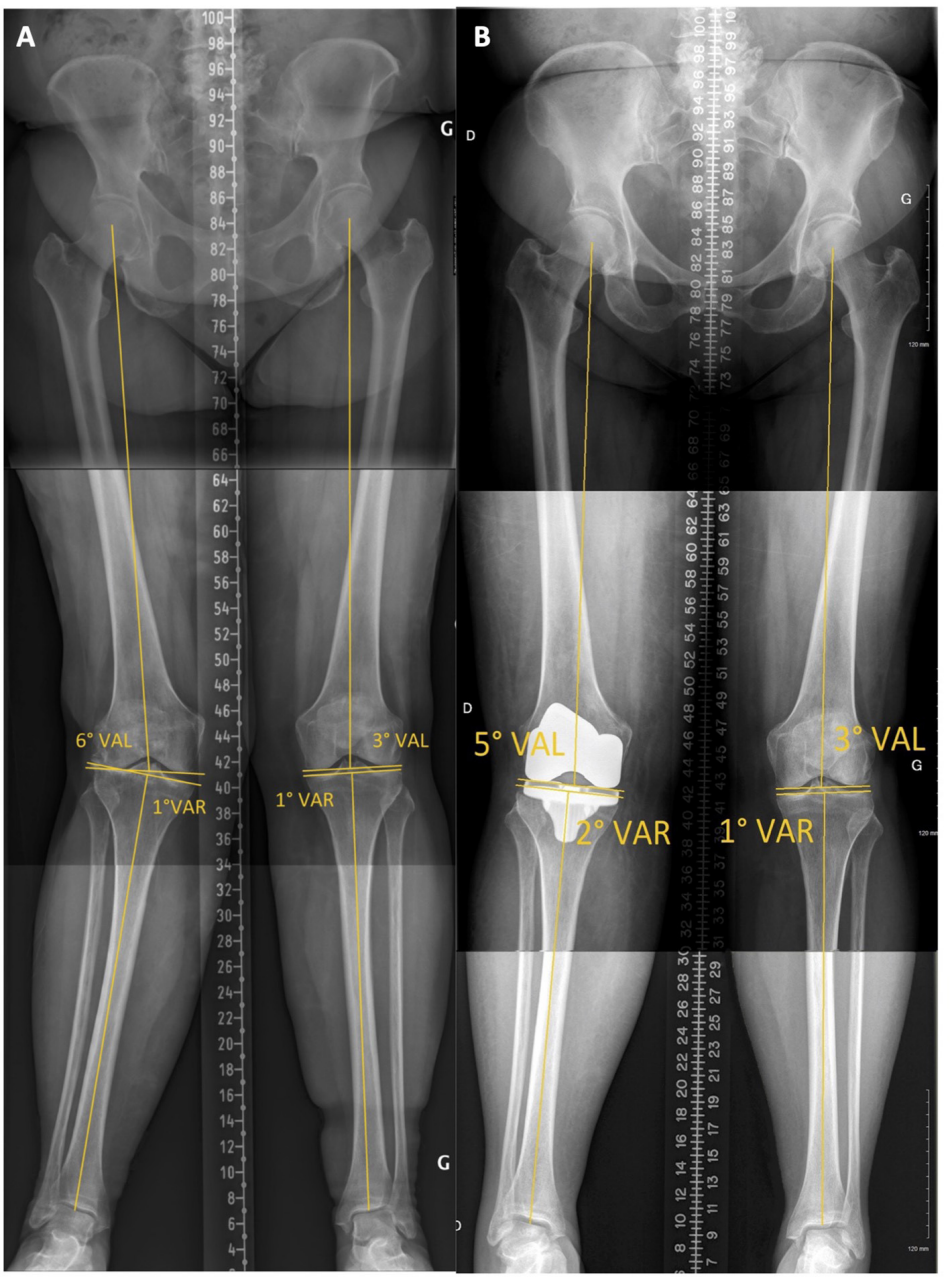
Case example 2, **(A)** preoperative standing long radiograph of a patient with a left lower limb mDFA of 6° valgus and a mPTA of 1° varus, resulting in an aHKA of 5° valgus. **(B)** on the postoperative radiograph, following the rKA algorithm, the implants mDFA is 5° valgus and mPTA is 2° varus.

A 79-year-old female with a painful osteoarthritic right knee with a valgus deformity (Figure 11A). The patient has an aHKA of 5° valgus, resulting from a mDFA of 6° valgus and mPTA of 1° varus. Following rKA algorithm (Figure 6), the mDFA of 6° valgus must be brought to 5° (Principle 2). To obtain an aHKA is <3° (Principle 1) and minimize femoral anatomy modification (Principle 4), further correction is required on the tibial side. A slight tibial varisation from 1° to 2° would lead to an acceptable aHKA of 3° valgus. We resurfaced the intact medial compartment (medial pivot point, Principle #5) and reduced lateral compartment resection thicknesses. Overall anatomy modification for this patient was 2° (1° on both the femur and tibia), and no ligamentous release was required to obtain satisfactory joint laxities. Postoperative radiograph confirms that we achieved our implant alignment goals using computer navigation (Figure 11B).

Further clinical examples are available in Video 4. https://youtu.be/GTi5me1tN4M. Surgeons should also understand how to manage specific/challenging cases (51, 52).

## Data Availability Statement

The original contributions presented in the study are included in the article/supplementary material, further inquiries can be directed to the corresponding author/s.

## Author Contributions

P-AV contributed to the conception and design of the method. WB wrote the first draft of the manuscript. P-AV, WB, and SM wrote sections of the manuscript. All authors contributed to manuscript revision, read, and approved the submitted version.

## Conflict of Interest

P-AV declares being a consultant for Microport, Stryker, Ethicon and Johnson & Johnson. The remaining authors declare that the research was conducted in the absence of any commercial or financial relationships that could be construed as a potential conflict of interest.
